# Temporal patterns of *broad *isoform expression during the development of neuronal lineages in *Drosophila*

**DOI:** 10.1186/1749-8104-4-39

**Published:** 2009-11-02

**Authors:** Baohua Zhou, Darren W Williams, Janet Altman, Lynn M Riddiford, James W Truman

**Affiliations:** 1 Department of Biology, University of Washington, Seattle, WA 98195, USA; 2 Department of Pediatrics, HB Wells Center for Pediatric Research, Indiana University School of Medicine, Barnhill Drive, Indianapolis, IN 46202, USA; 3 MRC Centre for Developmental Neurobiology, King's College London, Guy's Hospital Campus, London SE1 1UL, UK; 4 Janelia Farm Research Campus, Howard Hughes Medical Institute, Helix Drive, Ashburn, VA 20147, USA

## Abstract

**Background:**

During the development of the central nervous system (CNS) of *Drosophila*, neuronal stem cells, the neuroblasts (NBs), first generate a set of highly diverse neurons, the primary neurons that mature to control larval behavior, and then more homogeneous sets of neurons that show delayed maturation and are primarily used in the adult. These latter, 'secondary' neurons show a complex pattern of expression of *broad*, which encodes a transcription factor usually associated with metamorphosis, where it acts as a key regulator in the transitions from larva and pupa.

**Results:**

The Broad-Z3 (Br-Z3) isoform appears transiently in most central neurons during embryogenesis, but persists in a subset of these cells through most of larval growth. Some of the latter are embryonic-born secondary neurons, whose development is arrested until the start of metamorphosis. However, the vast bulk of the secondary neurons are generated during larval growth and bromodeoxyuridine incorporation shows that they begin expressing Br-Z3 about 7 hours after their birth, approximately the time that they have finished outgrowth to their initial targets. By the start of metamorphosis, the oldest secondary neurons have turned off Br-Z3 expression, while the remainder, with the exception of the very youngest, maintain Br-Z3 while they are interacting with potential partners in preparation for neurite elaboration. That Br-Z3 may be involved in early sprouting is suggested by ectopically expressing this isoform in remodeling primary neurons, which do not normally express Br-Z3. These cells now sprout into ectopic locations. The expression of Br-Z3 is transient and seen in all interneurons, but two other isoforms, Br-Z4 and Br-Z1, show a more selective expression. Analysis of MARCM clones shows that the Br-Z4 isoform is expressed by neurons in virtually all lineages, but only in those cells born during a window during the transition from the second to the third larval instar. Br-Z4 expression is then maintained in this temporal cohort of cells into the adult.

**Conclusion:**

These data show the potential for diverse functions of Broad within the developing CNS. The Br-Z3 isoform appears in all interneurons, but not motoneurons, when they first begin to interact with potential targets. Its function during this early sorting phase needs to be defined. Two other Broad isoforms, by contrast, are stably expressed in cohorts of neurons in all lineages and are the first examples of persisting molecular 'time-stamps' for *Drosophila *postembryonic neurons.

## Background

The sequential generation of distinct populations of neurons is a common feature across all taxa (for example, [1-3]). Nowhere is this more striking than in the development of the nervous system of insects having complete metamorphosis, such as *Drosophila*, where the central nervous system (CNS) must deal sequentially with the behavior of two radically different stages, the larva and the adult. This dichotomy is reflected in the proliferative activity of the neuronal stem cells (neuroblasts (NBs)) that produce the neurons for both larval and adult versions of the CNS. After the NBs segregate from the ventral ectoderm of the early embryo, they generate an initial set of neurons that form the larval CNS. This initial set includes a diverse array of neurons (for example, [4]) that establish the overall architecture of tracts and commissures, and provide the neural circuits that mediate larval behavior. The neuronal fates within an embryonic lineage are based on birth-order of the parent ganglion mother cells (GMCs) (for example, [5]). The molecular code defining birth-order is used across the embryonic lineages and is based on the sequential expression by the NB of the transcription factor genes *Hunchback*, *Kruppel*, *PDM*, and *castor *[1,6,7]. While present in the NB, each transcription factor is passed onto the GMC during mitosis and, subsequently, into the two daughter neurons when the GMC divides. These transcription factors encode different identities within the lineage and underlie the diversity of early neuronal types [7,8].

Following the generation of this diverse set of 'primary' neurons, the NBs produce a much larger set of cells during the extended phase of neurogenesis that occurs during larval growth. These 'secondary' neurons are morphologically more homogeneous than the primary set [9,10]. Studies on the K lineage in the moth *Manduca sexta *[11], as well as the lineages of the mushroom bodies [12] and antennal lobes [13] in *Drosophila*, show that the secondary neurons are generated in discrete blocks, with those in a block having similar properties. The molecular specification of these blocks is just beginning to be understood. After the expression of *castor *in the embryo, the NB expresses *grainyhead*, and this expression then persists through the remainder of the embryonic divisions and into the postembryonic phase of neurogenesis [14,15]. The BTB (broad-tramtrack-brick-a-brac) domain transcription factor Chinmo is expressed in neurons born during the early larval phase of neurogenesis and undergoes both transcriptional and translational regulation to establish the early γ neuron phenotype in the mushroom bodies and early projection neuron phenotypes in antennal lobe lineages [16]. More recently, the expression of *chinmo *was reported to be complementary to that of *broad *[17] (formerly called *Broad-Complex*), which encodes another BTB domain transcription factor.

Broad is best known for its role in metamorphosis and it plays a critical role in the proper transduction of ecdysone signals in different tissues during the larval-pupal transition (reviewed in [18]). Mutants null for *broad *show normal larval development but fail to initiate metamorphosis. Many *broad *mutations fall into one of three lethal complementation groups, and mutations in all three groups disrupt the metamorphosis of the CNS [19,20]. The *broad *gene encodes four major protein variants that share a common amino-terminal core region, including the BTB domain, but differ in their carboxy-terminal Zn fingers (Z1 to Z4) [21,22]. Using heat shock inducible cDNA transgenes, it was shown that the distinct genetic functions of the three lethal complementation groups could be ascribed to three of the Broad Zn finger isoforms (BR-Z1, -Z2, and -Z3) [22,23]. The function of the BR-Z4 isoform, though, remains unclear.

While most of the attention for the study of Broad has been directed towards its actions during metamorphosis, studies on the CNS suggest that this transcription factor has additional roles in development. Zhou [24] first reported the expression of Broad in the embryonic CNS of *Drosophila *and of the moth *Manduca*, and the maintenance of this expression through larval life. Recently, Maurange *et al*. [17] reported that the expression of Broad and Chinmo [16] divide the secondary neurons into two temporal groups at the start of metamorphosis. Here we show that it is the Br-Z3 isoform that complements Chinmo to set up this molecular dichotomy. We find, though, that Br-Z3 is transiently expressed in all interneurons, so that even the Chinmo+ neurons had expressed Br-Z3 earlier in larval life. The expression of both Chinmo [16] and Br-Z3 fades from the secondary neurons early in metamorphosis. However, two other Broad isoforms, Br-Z4 and Br-Z1, appear in a subset of neurons in every lineage to provide a molecular marker of neuronal birthdates that persists in the adult.

## Results

### Embryonic expression of *broad*

**Figure 1:**
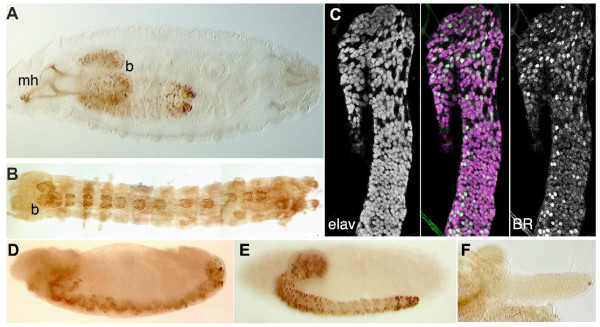
**Immunocytochemistry showing staining with a MAb directed against the core region of the Broad transcription factor**. **(A, B) **Whole mounts of *Drosophila *(A) and *Manduca *(B) embryos just prior to hatching. Broad expression is confined to the brain (b) and ventral CNS in both; mh, mouth hooks. **(C) **Confocal optical section through the thoracic and abdominal neuromeres of the CNS of a newly hatched larva of *Drosophila *that is double stained for the neuron-specific marker, Elav (magenta) and Broad (BR, green). All of the Broad-positive cells coincide with cells that also express Elav. Anterior is up; the CNS is slightly twisted so that only the right halves of the abdominal neuromeres are in the section. **(D, E) **Lateral views of *Drosophila *embryos at late stage 12 and stage 15, respectively, showing the expression of Broad confined to the developing CNS. **(F) **The CNS of a newly hatched larva lacking *broad *function (*br*^*npr*3 ^homozygote) showing no Broad immunostaining.

Individuals lacking *broad *function are normal through larval growth [25,26], and Northern blot analyses first show *broad *transcripts in the last larval stage in preparation for metamorphosis (for example, [27]). Consequently, we were surprised when we found that the monoclonal antibody (MAb) directed against the Broad core domain showed prominent immunostaining in *Drosophila *embryos. Control embryos that were homozygous for the null allele *br*^*npr*3 ^[26] showed no Broad immunoreactivity (IR; Figure 1F), confirming that the wild-type pattern was, indeed, due to Broad.

Embryonic Broad expression was confined to the CNS (Figure 1A). It first appeared in late stage 12 embryos, just before completion of germ band retraction (Figure 1D), and remained high through the remainder of embryogenesis and through hatching (Figure 1A, E). Staining with isoform-selective antisera showed that the early larval CNS immunostaining was due to the Br-Z3 isoform. *In situ *hybridization studies using probes directed against zinc finger regions of each of the various isoforms also confirmed that only the Br-Z3 isoform was expressed at this time (data not shown).

Double staining of hatchling nervous systems with antibodies against the neuron-specific protein Elav [28] and against Broad-core showed that Broad was expressed only in neurons (Figure 1C). Fluorescence immunocytochemistry detected weak Broad-IR in most neurons, but scattered cells showing strong expression were concentrated in the brain and thoracic and terminal abdominal neuromeres. Strongly expressing neurons were especially obvious using horseradish peroxidase-conjugated secondary antibodies that were visualized with diaminobenzadine (for example, Figure 1A, E). No Broad-IR was seen in the embryonic peripheral nervous system (Figure 1A, D, E).

The expression of Broad in the embryonic and larval CNS may be a general feature of holometabolous larvae. As seen in Figure 1B, first instar larvae of the tobacco hornworm moth *M. sexta *also showed prominent Broad-IR in their CNS. As in *Drosophila*, Broad expression was restricted to neurons. Other tissues in *Manduca *start to express Broad only late in the last larval instar, in preparation for metamorphosis [29].

### Broad expression in embryonic secondary neurons

To identify the strongly Broad-expressing neurons seen in the embryo and early larva, we first looked for Br-Z3 expression in a number of identified motoneurons and modulatory neurons, including those containing octopamine, crustacean cardioactive peptide, FMRFamide, and eclosion hormone. All were negative for Br-Z3 (data not shown). The greatest numbers of Broad+ neurons were in regions of the CNS that contained dormant embryonic NBs that would later reactivate for a second round of neurogenesis during larval growth [30,31]. Consequently, the Br-Z3 positive neurons might be related to the large lineages that include both embryonic and larval progeny. During their lifetime, most NBs generate an initial set of highly diverse neurons, the primary neurons, followed by a much larger set of more homogeneous cells, the secondary neurons [9,32]. While primary neurons immediately differentiate to make the larval CNS, the secondary neurons typically arrest shortly after their birth and wait until metamorphosis before elaborating their dendritic and axonal arbors. Most secondary neurons are born during the larval phase of neurogenesis but a few are thought to be born in the embryo prior to the embryonic arrest of their NB. They are readily identifiable in the larva because of their scant cytoplasm and lack of branching [9,33], and they are concentrated in the thoracic neuromeres and the brain, a distribution similar to the Br-Z3 positive neurons.

**Figure 2:**
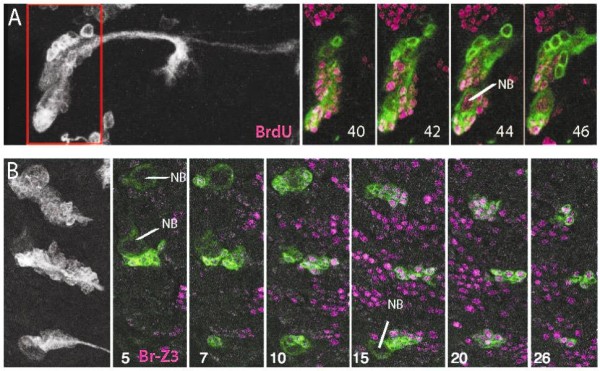
**Examples of early embryonic induced MARCM clones and showing embryonic-born secondary neurons and their state of *Broad *expression**. Left: Z-projections of green fluorescent protein (GFP)-labeled clones. Right: individual confocal sections at various levels through the clone (section number) showing GFP-labeled neurons (green) and either BrdU or Br-Z3 (magenta). **(A) **Early embryonic induced neuroblast clone of thoracic lineage 9 from a third instar larva that had fed on a BrdU diet from the time of hatching. The large basal neurons in the clone lack BrdU incorporation, showing that they were born before hatching. The red-framed area is represented in the individual sections. **(B) **Three neuroblast clones in the lateral thorax at the start of the third larval instar (approximately 72 h after egg laying), showing that the basal neurons express Br-Z3 at this time. NB, neuroblast.

To examine the arrested secondary neurons, we induced early embryonic MARCM (mosaic analysis using a repressible cell marker) clones using the *elav*^*C*155^*GAL4 *driver. In the context of MARCM, the *elav*^*C*155^*GAL4 *driver expresses prominently in the arrested, secondary neurons [9]. Embryonic versus postembryonic neurons in NB clones were distinguished by feeding larvae on a diet containing bromodeoxyuridine (BrdU) from hatching [30], thereby labeling all of the neurons born during the larval phase of neurogenesis. Figure 2A shows an example of a lineage 9 NB clone from a larva that was fed a BrdU diet from hatching. All of the neurons in this clone had the appearance of arrested, secondary neurons showing the extension of an initial neurite, but lacking interstitial or terminal branching. The most basal neurons in the clone showed no BrdU incorporation, showing that they were born prior to hatching. Figure 2B shows an example of Br-Z3 expression in clones induced at the same time and examined at the start of the third larval instar (approximately 72 h after egg laying (AEL)). All of the basal neurons were Br-Z3 positive, whereas the most apical neurons had not yet begun to express Broad. Combined with the BrdU results, we conclude that arrested, embryonic-born secondary neurons are among the population of Br-Z3 positive cells seen at hatching.

### Br-Z3 expression in postembryonic born neurons

#### Secondary neurons of the brain and thoracic lineages

The number of Br-Z3-expressing neurons was stable until the start of the third larval instar, when the number in the central brain and thoracic neuromeres then started to increase. The following description of Br-Z3 expression focuses on the secondary neurons born during the larval phase of neurogenesis. MARCM clones were induced after hatching (24 to 26 h AEL) and then examined at various times thereafter. There is a repeating set of 25 NBs (numbered 0 to 24) in each thoracic hemisegment of the larval CNS. Clones arising from each NB can be unambiguously identified in the larva by their position in the cellular rind and by the trajectory through the larval neuropil of the bundled neurites that emerge from the cluster [9,34].

**Figure 3:**
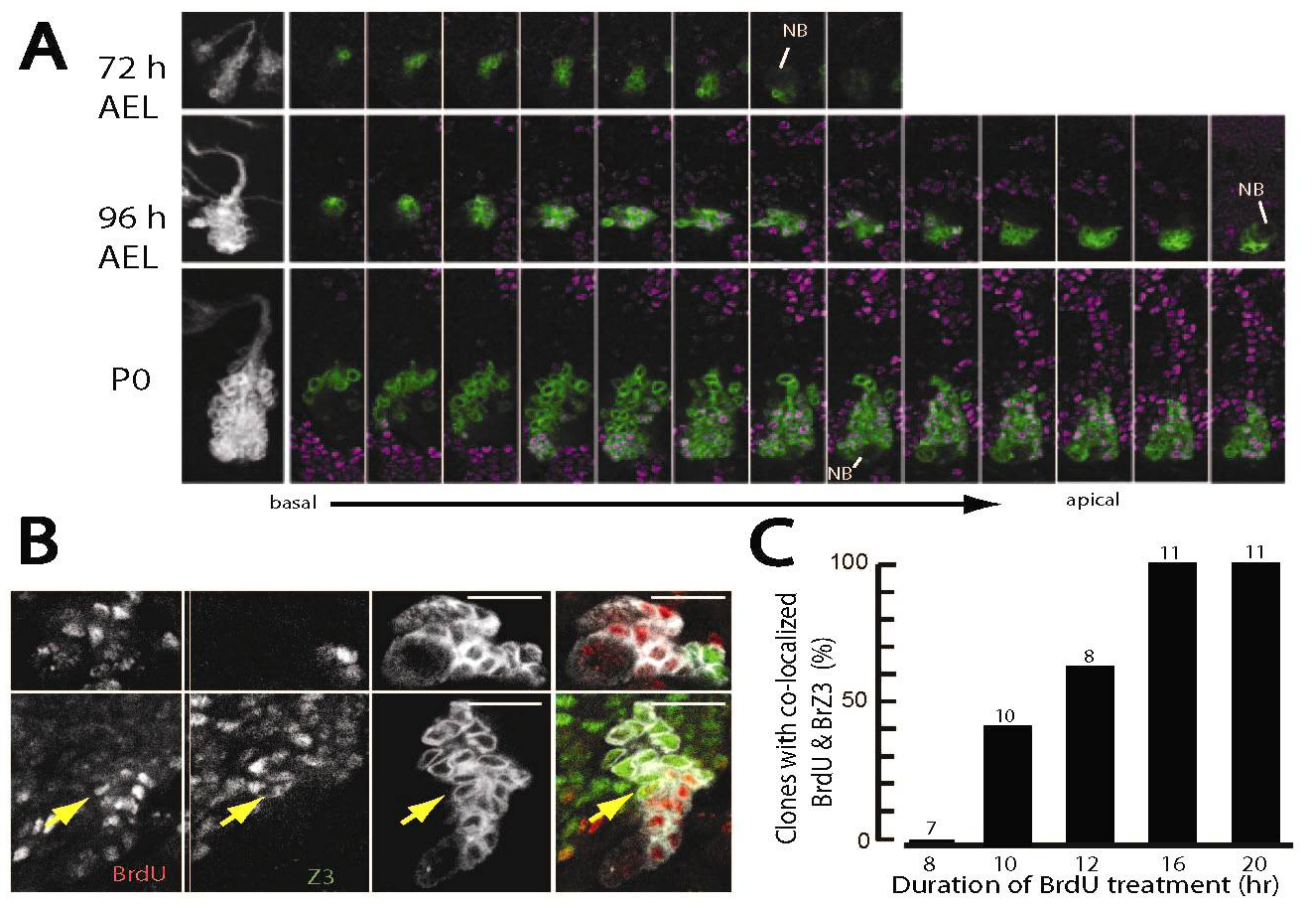
**The time-course of Br-Z3 expression in neuroblast MARCM clones induced after hatching**. **(A) **Three examples of thoracic neuroblast clones for lineage 3 showing the change in Br-Z3 expression in neuronal populations through larval growth. Left: Z-projection of the clones showing the increase in overall size and morphology at 72 and 96 h AEL, and at puparium formation (P0). Right: sequential sections through each clone showing the distribution of Br-Z3+ neurons in the cluster. At 72 h AEL, Br-Z3 is barely detectable in larval born neurons; by 96 h AEL the basal cells (left) are Br-Z3+ but the more apical cells are negative. At P0 the basal cells (left) are now negative while more apical cells are Br-Z3+ except for those nearest the neuroblast (NB). **(B, C) **Use of BrdU pulses at 96 h AEL to establish the time of Br-Z3 expression relative to the end of DNA synthesis in the parent GMC. (B) Confocal sections through neuroblast clones for lineage 3 from larvae fed on a BrdU diet for 8 (top) or 16 h (bottom) prior to sacrifice. Br-Z3- and BrdU-labeled cells did not overlap after the 8 h pulse but did so after the 16 h pulse. Arrow points to the same nucleus in each panel. (C) The frequency of clones that had co-labeled cells as a function of the duration of the BrdU pulse. Numbers above each bar are the numbers of clones examined for each treatment.

Figure 3A shows examples of the lineage produced by thoracic NB 3. The thoracic NBs resume dividing around 48 h AEL [30], and the 72-h, lineage 3 clone contained 24 cells along with the NB. Very weak Br-Z3 expression was seen in only the most basal (oldest) cells in the cluster. The lineage 3 clone from 96 h AEL contained 78 neurons. The basal neurons were Br-Z3 positive, while the youngest cells, nearest the NB, were negative. The example of lineage 3 at pupariation had 183 cells. The shape of the clone was somewhat distorted by the growing CNS, but the larger neurons near the neuropil had lost their Br-Z3 expression. The remaining cells were strongly Br-Z3 positive with the exception of those nearest the NB. The pattern of Br-Z3 expression described for lineage 3 was typical for all of the lineages of thoracic interneurons. Unlike the MARCM clones induced in the early embryo, which had some basal cells that expressed prominent levels of Br-Z3 when examined at 72 h (Figure 2B), clones that contained only postembryonic-born neurons had just traces of Br-Z3 evident at this time. By 96 h AEL, though, Br-Z3 was expressed strongly in all neurons in the clone except for the youngest, but it then disappeared from the basal neurons by the time of wandering and pupariation.

Unlike the interneuron lineages, the two motor lineages, from NBs 15 and 24, showed little Br-Z3 expression at any time. We occasionally saw weak Br-Z3 in the two youngest lineage 15 motoneurons in wandering larvae, but more frequently, we saw nothing (data not shown).

The Br-Z3 negative cells nearest the NB include both GMCs and young neurons. Expression of membrane-localized Notch is a feature of the NB, GMCs and the youngest neurons [35] and we find that Notch and Br-Z3 expression are mutually exclusive (JA and JWT, in preparation). We determined the timing of Broad appearance in newborn interneurons by using 96 h AEL larvae that had MARCM clones that had been induced after hatching. They were given food containing BrdU for various periods and then examined for the overlap of BrdU and Br-Z3 immunostaining in the clones. Figure 3B gives a specific example for lineage 3; after 8 h on BrdU food none of the labeled cells expressed Br-Z3, but at 16 h we consistently found BrdU-labeled cells that also expressed Br-Z3. About 11 h on a BrdU diet was needed to have co-labeled neurons in 50% of the clones (Figure 3C). The significance of this timing will be considered in the Discussion.

**Figure 4:**
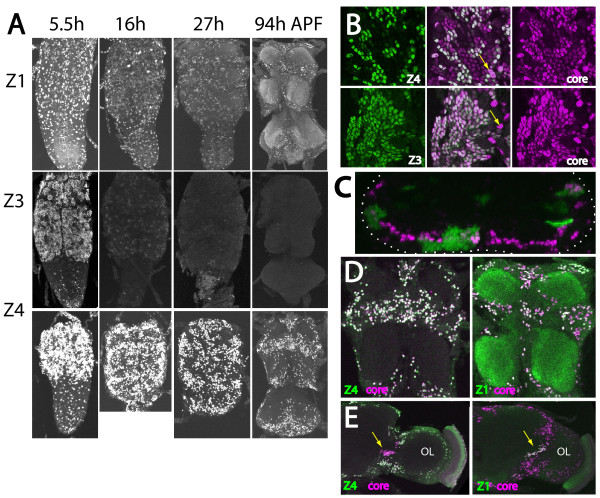
**Dynamics of Broad isoform expression during metamorphosis**. **(A) **Z-projection of the ventral CNS showing the distribution of nuclei expressing Br-Z1, -Z3 and -Z4 at various times after puparium formation (APF). The neuropil staining for Br-Z1 at 94 h APF is likely due to a cross-reacting epitope rather than Broad. **(B) **Confocal sections showing Broad immunostaining in secondary neurons in the apical layers of the thoracic neuromeres at puparium formation. Br-Z4+ neurons are a subset of neurons that show Broad-core immunostaining. The Br-Z3+ neurons completely overlap the Broad-core immunostained neurons. The large nuclei of glia cells show no Br-Z3 and reduced Br-Z4 (arrows). **(C) **Cross-section in the T2 region of the CNS from a wandering stage larva that carried green fluorescent protein (GFP)-labeled MARCM clones and was immunostained for Br-Z4. Br-Z4+ cells were located at an intermediate layer of the cellular rind; the dotted line shows the outline of the CNS. **(D, E) **Comparison of immunostaining for Br-Z4 and Br-Z1 with that of Broad-core in the pharate adult nervous system. (D) Ventral view of the T1-T2 neuromeres showing that essentially all of the Broad-core+ neurons also show Br-Z4 immunostaining. By contrast, only a moderate number of Broad-core neurons also show the Br-Z1 epitope. (E) The brain shows a cluster of neurons that express BR-Z1 but not Br-Z4 (arrow) near the junction with the optic lobes (OL).

For most of the secondary neurons, Br-Z3 expression was maintained through the start of metamorphosis. It was still evident at 5.5 h after puparium formation (APF) but by 16 h APF it was barely detectable and, then, only in the youngest neurons in each cluster (Figure 4A). This isoform was completely gone by 27 hours APF (Figure 4A).

#### Mushroom bodies

**Figure 5:**
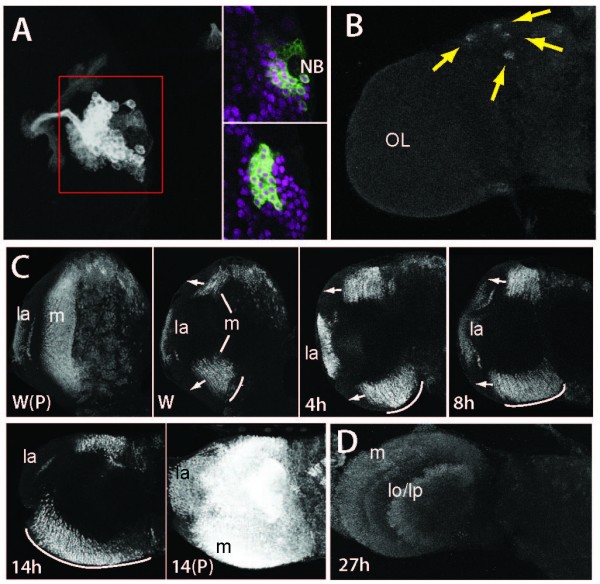
**Br-Z3 expression in specialized brain regions**. **(A, B) **Expression of Br-Z3 in neurons of the mushroom bodies. (A) A projected confocal z-stack showing an embryonically induced MARCM clone in the mushroom bodies of a mid third instar larva. Two individual sections from the red-framed area are on the right and show the expression of Br-Z3 (magenta) in the green fluorescent protein (GFP)+ (green) cells of the clone. Only the neuroblast (NB) and its most recently born progeny lack Br-Z3 expression. (B) A projected confocal Z-stack of a pharate adult brain (95 h APF) showing the four small clusters of Br-Z3 expressing neurons in the mushroom bodies (arrows). OL, optic lobe. **(C, D) **The changes of Br-Z3 expression in the optic lobe through early metamorphosis. (C) The progression of Br-Z3 in the neurons of the optic lobe. The confocal images at late wandering show a complete Z-projection (W(P)) and a confocal single slice (W). Br-Z3 expression is strong in the lamina (la) and medulla (m) but only the neurons in the most medial columns of medulla express Br-Z3. Subsequent sections show recruitment of more lateral medulla columns (arrows) in older prepupae (hours APF) with the inclusion of all of the medulla neurons by 14 h APF. 14(P): a full Z-projection of the 14 h preparation. In the sections, the arc indicates the extent of Br-Z3 expression in the medulla. (D) Br-Z3 expression in a confocal Z-projection at 27 h APF. Br-Z3 expression is still present in neurons of the medulla and lobula and lobula plate (lo/lp) even though it has disappeared from all other regions of the CNS except the mushroom bodies.

At hatching, the embryonic-born neurons of the mushroom bodies showed strong expression of Br-Z3 (data not shown). The status of Br-Z3 expression in later-born cells was determined by inducing MARCM clones at hatching and then examining Br-Z3 expression in the clones at various times thereafter. At 72 h AEL, all mushroom body neurons in the clone showed prominent Br-Z3 expression except for a small number of cells nearest the NB (Figure 5A). Clones examined at 96 h showed the same pattern with a marked increase in the number of Br-Z3 positive cells and a small apical cell cluster that was negative. This pattern continued to pupariation (data not shown).

Br-Z3 expression was lost from most mushroom body neurons by 18 h APF. However, neurogenesis continues in the mushroom bodies almost up to the time of adult emergence [30]. Accordingly, we continued to find Br-Z3 expression in four small clusters of neurons in each mushroom body through the remainder of metamorphosis up to the time of adult emergence (Figure 5B, arrows). Each cluster was in close proximity to a mushroom body NB. This spatial relationship suggests that recently born mushroom body neurons show transient Br-Z3 expression regardless of when they were born during metamorphosis.

#### Optic lobes

Figure 5C shows the progression of Br-Z3 expression in the optic lobes. Br-Z3 was expressed by young neurons of the lamina, medulla and lobula. The layered structure of the medulla, though, made the pattern of Br-Z3 expression the easiest to track through time. Br-Z3 was first seen in medulla neurons in the mid-third instar larva soon after they had started to be born (data not shown). The expressing neurons formed columns, with the most medial columns being Br-Z3 positive. By late wandering, expression was seen in approximately 15 columns (Figure 5C). With time after pupariation the appearance of Br-Z3 progressively spread into more lateral columns (Figure 5C, arrows), so that by 14 h APF the entire medulla was Br-Z3 positive. Strong expression of Br-Z3 was still evident in the optic lobes through 27 h APF (Figure 5D), well after it had disappeared from the rest of the CNS, but it was gone by 48 h APF (data not shown).

### Br-Z4 and Br-Z1 are markers for neuronal birthdates

The Br-Z1 and Br-Z4 isoforms appeared in the CNS at the start of metamorphosis. Glia showed prominent expression of Br-Z1 and -Z4 at pupariation (data not shown) and at 5.5 h APF (Figure 4A), but glial expression had disappeared by 27 h. As previously reported by Consoulas *et al *[36], we did not find the reappearance of any Broad isoforms in the functional larval neurons, which would either die or remodel. Some secondary neurons, however, showed Br-Z4 in addition to Br-Z3.

The Br-Z4 expression was evident in some secondary neurons by the onset of wandering (data not shown). A number of features of Br-Z4 expression were intriguing. Firstly, Br-Z4 appeared in all of the thoracic secondary lineages (except the small motor lineage, lineage 24), but in only a subset of neurons in each. Secondly, the Br-Z4 positive neurons were clustered in each lineage, suggesting that they arose from consecutive GMCs. Thirdly, at pupariation, the Br-Z4-expressing neurons were concentrated at an intermediate level in the cellular rind (Figure 4C), suggesting that they were born at similar times across the lineages. Indeed, this layering in the cellular cortex is similar to that seen in the embryo for the transcription factors that establish the temporal identities of neurons during embryogenesis [6].

An indication of the temporal origins of the Br-Z4+ neurons came by comparing the immunostaining against the isoform-specific Z3 and Z4 zinc fingers versus against the core region of Broad (Figure 4B). Maurange *et al *[17] showed that at pupariation the secondary neurons are divided into an older group of Chinmo+ neurons and a younger set that express Broad-core-IR. As seen in Figure 4B, all of the Broad-core positive neurons also showed immunostaining for the Z3 zinc finger, showing that they all express Br-Z3. Large, Broad-core positive but Z3-negative nuclei having prominent nucleoli (Figure 4B, arrows) were immunopositive for *repo *(data not shown), showing that they are larval glia [37]. The Br-Z4+ neurons were a subset of the Broad-core-IR cells and, therefore, not members of the group of older, Chimno+ cells.

**Figure 6:**
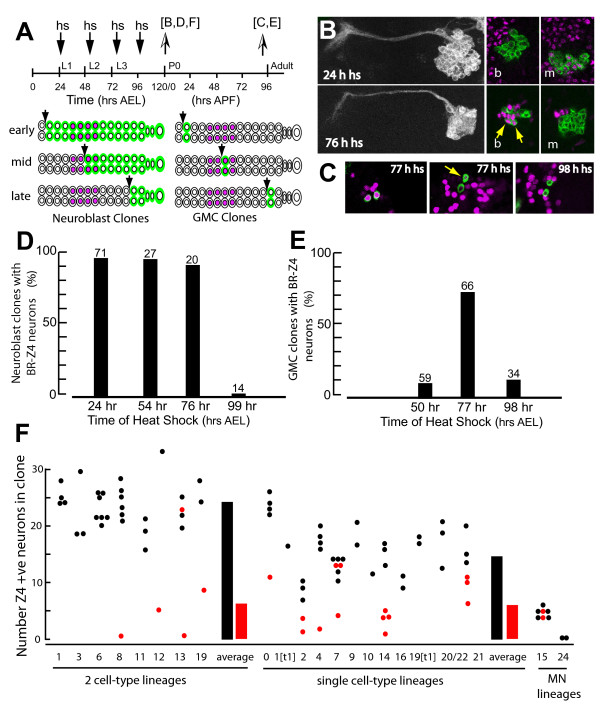
**Time of birth and distribution of Br-Z4+ neurons in the thoracic lineages**. **(A) **Schematic, showing the timing of heat-shocks (hs; arrows) to induce MARCM clones during larval neurogenesis and the time of analysis of the clones at pupariation [B, D, F] or just before adult emergence [C, E]. The cartoons show the predicted relationship of clone boundaries (green) to Br-Z4 expression (magenta), if the Br-Z4+ neurons were born during a discrete phase of larval life. P0, puparium formation. **(B) **Z-projections of lineage 14 neuroblast clones that were examined at pupariation but induced by heat shocks at 24 h or 76 h AEL. Left: anatomy of the whole clone. Right: sections from the basal (b) and middle (m) portions of the clone. The basal cells in early induced clones are Br-Z4 negative with the Br-Z4+ cells clustered in the middle layers. In the 76 h-induced clone, the oldest (basal) cells included only a few Br-Z4+ neurons (arrows) but positive neurons were outside the clone. **(C) **Examples of two-cell GMC clones from the adult CNS; examples from heat-shocks at 77 h AEL show both sibs expressing Br-Z4 or one sib with weak expression (arrow) and the other negative. Both sibs are negative after the late heat-shock induction. **(D) **Summary of neuroblast MARCM clones that were examined around the time of pupariation. Only clones induced at 99 h AEL were completely devoid of Br-Z4+ neurons. **(E) **Summary of GMC clones that were examined just prior to adult emergence. Br-Z4+ neurons were most commonly seen in clones induced at 77 h AEL. For (C, D), the numbers above each bar are the numbers of clones examined. **(F) **Counts of the number of Br-Z4+ neurons in neuroblast MARCM clones that were induced at 24 h (black dots) and 76 h (red dots) AEL. The neuroblast of origin was identified by the morphology of the axon bundles that exited the clone. Interneuron-generating lineages were grouped by whether they produced one or two classes of interneurons. Some neuroblasts (NBs 1 and 19) were in both categories because of segment-specific loss of one class of interneurons. The black and red bars give the averages for the two groups. MN, motor neuron lineages.

To better determine when the Br-Z4 positive neurons were born, we induced MARCM clones at various times during larval life and counted the number of Br-Z4 positive neurons in NB clones at pupariation (Figure 6A). Figure 6F shows the results from 69 clones induced soon after hatching. This sample included 21 of the 25 thoracic lineages. As illustrated by lineage 14 (Figure 6B), the most basal and apical cells were Br-Z4 negative and Br-Z4+ cells were in the middle of the cluster. Nine of the thoracic NBs produce two major classes of interneurons, while 14 NBs produce only one interneuron class [9]. The former lineages had an average of 26 Br-Z4-expressing neurons, whereas the latter averaged 16 neurons (Figure 6F). The remaining two thoracic NBs make only motoneurons: NB 24 makes about eight motoneurons and none were Br-Z4+, whereas NB 15 makes 26 to 28 motoneurons, of which four to five were Br-Z4+.

Clones induced at 54 h AEL showed numbers of Br-Z4+ neurons that were similar to those seen in clones induced at hatching (data not shown). However, clones induced at 76 h AEL typically had a reduced number of Br-Z4+ neurons (Figure 6F). As seen for the lineage 14 example in Figure 6B, the typical pattern for the 76 h clones was for the Br-Z4+ neurons to be at the base of the clone and clustered with Br-Z4+ cells outside the clone boundary. These late induced clones had an average of only six Br-Z4+ cells in the clone. Only in clones for lineages 7 and 13 did we find numbers of BR-Z4+ cells that were similar to that seen in the early-induced clones. These higher values may reflect some variability amongst the lineages as to when their Br-Z4 cells are born. However, the overall pattern indicates that GMCs that are born around 76 h AEL make the neurons that will subsequently express Br-Z4. The larger motor lineage, lineage 15, had only a few Br-Z4+ cells and these all appeared to be born after 76 h. We found no Br-Z4+ cells in clones induced at 99 h AEL and examined at pupariation (Figure 6D).

Broad was prominently expressed in most secondary neurons at the start of metamorphosis, with most of the neurons expressing Br-Z3 and about 20% expressing both Br-Z3 and Br-Z4 (Figure 4B). As metamorphosis progressed, the Br-Z3 was lost from all of the secondary neurons but Br-Z4 expression persisted (Figure 4A). Consequently, by the time of adult emergence the only cells that still expressed Broad were expressing the Br-Z4 isoform (Figure 4A, D), with the minor exceptions noted below. To determine if the neurons that expressed Br-Z4 in the adult were the same cells that were expressing this isoform at the start of metamorphosis, we induced MARCM clones at various times during larval life and then examined expression of Br-Z4 just prior to adult emergence. We scored expression in GMC clones because these would identify neurons born in the window shortly after the heat-shock (Figure 6A). As shown in Figure 6E, Br-Z4+ cells were rarely observed in GMC clones induced at 50 h, were common in clones induced at 77 h, and rare again in 98 h clones. This temporal pattern of Br-Z4 expression is consistent with that in NB clones analyzed at pupariation (Figure 6D) and indicates that the neurons expressing Br-Z4 at pupariation maintain their expression into the adult. For two-cell GMC clones generated around 76 h, both siblings were usually Br-Z4+, but we sometimes also found examples of a weakly expressing sib along with one that was negative (Figure 6C). In late induced clones, the sibs were Broad-Z4 negative (Figure 6C).

The expression of Br-Z1 was evident by 16 h APF and also remained in some neurons into the adult (Figure 4A). The number of Br-Z1+ cells in a lineage was much smaller than the number of Br-Z4+ neurons. We compared Br-Z1 and Br-Z4 expression to that of Broad-core immunostaining in flies just before adult eclosion (Figure 4D, E). In the thoracic neuromeres, the neurons that had Broad-core immunostaining were also positive for Br-Z4. By contrast, Br-Z1+ cells made up only a small subset of the Broad-core+ neurons (Figure 4D). This pattern suggests that most of the Br-Z4+ neurons express this isoform alone, but a few neurons in each cluster express both Br-Z1 and Br-Z4. We do not know if Br-Z1 expression is characteristic for neurons born during a specific phase of the Br-Z4 window.

Although Br-Z1 was usually expressed along with Br-Z4, we found one notable example in which Br-Z1 was expressed alone. A large cluster of neurons at the juncture between the optic lobes and the brain showed strong Br-Z1 expression in the absence of detectable Br-Z4 (Figure 4E, arrow). We do not know the identity of these interneurons.

### Effects of ectopic expression of Br-Z3

**Figure 7:**
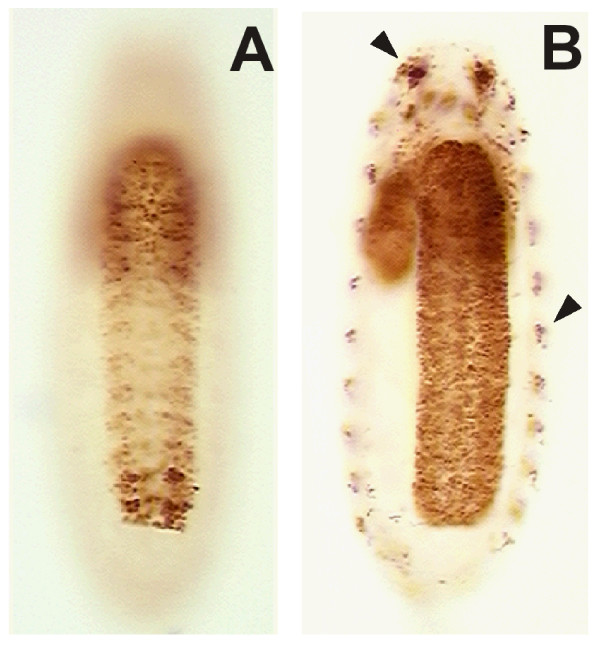
**Ectopic expression of Br-Z3 in all neurons in stage 17 embryos as revealed by immunostaining with the Broad-core MAb**. **(A) **Wild-type Broad expression seen in the CNS of a late embryo carrying a *UAS-Br-Z3 *transgene (line *UAS-Br-Z3*^216-1^). Ventral expression is primarily in the thoracic and terminal abdominal neuromeres. **(B) **Broad expression in a late embryo of the genotype *elav*^*C*155^; *UAS-Br-Z3*^216-1^. Broad is expressed throughout the CNS as well as in the peripheral nervous system (arrowheads).

Since mutants null for *broad *display no obvious larval phenotype, we wanted to determine if *broad *misexpression would generate a phenotype. We initially used *elav*^*C*155^*GAL4 *to drive *UAS-Br-Z3*. This resulted in Br-Z3 being widely expressed in the embryonic CNS and peripheral nervous system (Figure 7). Four independently transformed *UAS-Br-Z3 *lines were used in these experiments and, while differing in hatching rate, they gave similar phenotypes.

The larvae that hatched showed normal morphology but sluggish movements. Most failed to feed and died during the first instar. Some less severely affected larvae could feed and eventually molted to the second instar. The movements of these second instar larvae were more severely affected than those of the first instar larvae: they showed little coordinated movement, although their muscles were capable of contraction. The most severe phenotypes were obtained from the cross of *elav*^*C*155^*GAL4 *and *UAS-Br-Z3*^216-1^. Less than 1% of the larvae hatched and these showed reduced crawling, even after stimulation. The unhatched embryos from all the crosses showed no gross anatomical defects.

**Figure 8:**
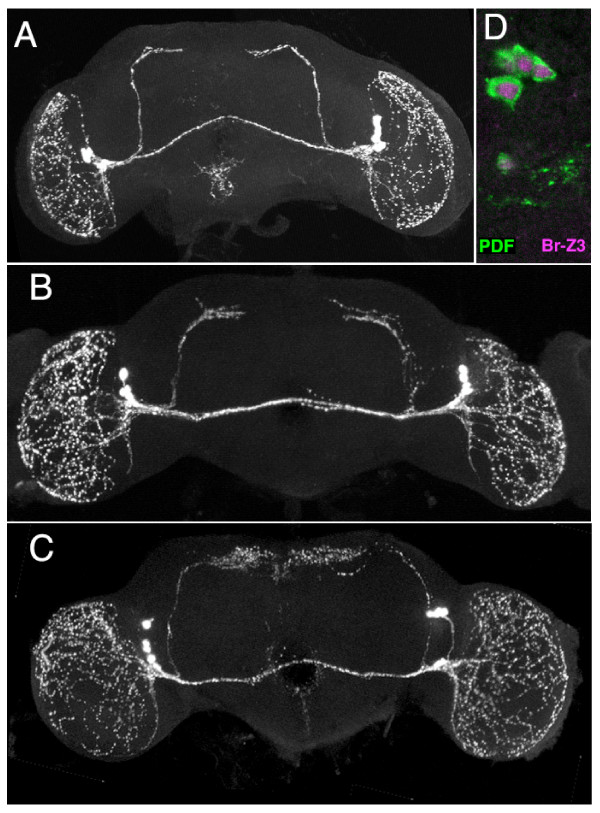
**Projected confocal z-stacks of adult brains showing the effect of targeted expression of Br-Z3 on the metamorphic growth of the pigment dispersing factor-containing neurons that project to the dorsal protocerebrum**. Images are frontal views of pigment dispersing factor (PDF)-stained neurons. **(A, B) **The medial projections of the small pdf neurons in *pdf-GAL4 *(A) and *UAS-Br-Z3 *(B) resembled that of wild-type, showing a simple termination well before the midline. **(C) **The pdf neurons of a *pdf-GAL4*; *UAS-Br-Z3 *individual showing the extensive branching of their terminals towards the midline. **(D) **Confocal section from a *pdf-GAL4*; *UAS-Br-Z3 *adult showing strong ectopic expression of Br-Z3 (magenta) in the PDF-expressing neurons (green).

A more selective expression of Br-Z3 was accomplished using a *pdf-GAL4 *line to drive expression of both *UAS-Br-Z3 *and *UAS-mCD8::GFP *in the large and small LN_v _neurons in the brain [38]. The constitutive expression of Br-Z3 in these cells had no obvious effect on their larval morphology (data not shown), but there were clear effects at metamorphosis (Figure 8). The small LN_v _neurons project to the dorsal protocerebrum and persist into the adult. At metamorphosis, they prune back their axon and then elaborate a compact projection that terminates well before the midline. The normal projection pattern was seen in both parental lines (Figure 8A, B), but Br-Z3 expression in these neurons resulted in a diffuse spray of neurites that extended to the midline (Figure 8C). The enhanced sprouting of the Br-Z3-expressing neurons was evident from the beginning of outgrowth at about 24 h APF (data not shown). This exuberant growth was unique to the expression of the Br-Z3 isoform and not seen when the other three Broad isoforms were individually expressed in these cells (data not shown).

## Discussion

### Broad isoforms and central nervous system metamorphosis in *Drosophila*

In *Drosophila *and other insects with complete metamorphosis, *broad *expression is associated with the initial phase of metamorphosis - the transition of the larva to the pupa. The *broad *isoforms play critical roles in this transition and are needed for the proper 'read out' of ecdysone-coordinated molecular events (reviewed in [18]). In *broad *null mutants (for example, *br*^*npr*3^) the premetamorphic growth and development of larval and imaginal tissues appear normal, but metamorphosis is blocked in all tissues before puparium formation [25,26]. In *br*^*npr*3 ^mutants, the larval CNS appears to be normal before the wandering stage, although its subsequent metamorphosis is disrupted [19].

While the classic role for *broad *is in the regulation of metamorphosis, the gene is known to have a wider role in development. It is involved in chorion formation during oogenesis in adult *Drosophila *(for example, [39]), and here we show that Br-Z3 expression is initiated in the CNS of the late embryo and that Br-Z1 and Br-Z4 continue to be expressed in the adult. The origins of the four isoforms of *broad *are ancient, extending back into the Crustacea [40], much earlier than the evolution of complete metamorphosis in insects. Functions typified by its role in the nervous system may represent an ancestral function for *broad*, and its role in metamorphosis a more recently derived function for this gene.

#### Primary and secondary neurons

In the developing vertebrate CNS, and especially the spinal cord, the initial set of neurons that set-up the basic architecture of the CNS are often called primary neurons. They are typically large and readily identifiable [41] and may be later replaced by a larger flood of secondary neurons. In metamorphic species, like frogs, the primary neurons are ascribed to function in the larval tadpole while secondary neurons are generated in preparation for metamorphosis to the adult frog [42]. An analogous concept of primary and secondary neurons is becoming established for *Drosophila *development. Similar to that seen in metamorphic vertebrates, the term primary neuron is typically associated with cells born during the first phase of neurogenesis in the embryo and secondary neuron for the cells born during the later, larval phase (for example, [43]). The larval-born cells arrest their development soon after their birth and wait until metamorphosis before maturing into functional neurons. However, our BrdU feeding experiments (Figure 2A) show that some embryonic-born neurons also arrest without showing any branching, a condition identical to that seen in the neurons that are born in the larva. Moreover, it appears that these arrested, embryonic neurons show a sustained expression of Br-Z3, a feature characteristic of the secondary neurons born during the larval phase of neurogenesis.

In light of the latter observations, it is worth contemplating the definition of primary and secondary neurons. There are three potential uses of this designation/terminology. One would be to use it to denote embryonic versus postembryonic born neurons. A second would be to use it to refer to the functional status of a neuron, where embryonic neurons that are fully differentiated, functional components of a network could be termed primary neurons and neurons that had initiated neurite growth but 'stalled' their development prior to establishing pre- and postsynaptic specializations would be secondary neurons. A third use would be to refer to the developmental transition within lineages where a neural precursor switches from producing diverse progeny at each division to generating homogenous 'blocks' of cell types.

There is a striking difference between the mechanisms of specification of neuronal identities of early and late-born neurons in insects. For a given NB, the characteristics of the first few progeny can be strikingly diverse and are based on birth-order of the parent GMC. The determination of birth-order is based on unique molecular identities that are bestowed on successive GMCs through an intrinsic, temporal program of transcription factor production by the NB and the stable passage of that expression to the GMC during cell division [6]. A series of loss-of-function and gain-of-function studies confirmed that the presence of these transcription factors is responsible for the diversity of neuronal phenotypes within a given lineage and underlies the great diversity of early neuronal types seen across the lineages [7,8]. This intrinsic progression, however, appears to stop after the expression of *castor*, and remaining neurons, both embryonic and postembryonic, express the transcription factor gene *grainyhead *[14,15]. During the postembryonic neurogenic period, the NBs typically produce groups of neurons with similar characteristics. This is most evident during the postembryonic phase of neurogenesis in moths [11] and flies [12,13], where we see neurons being produced in discrete blocks of similar cells, rather than as unique individuals. The expression of Chinmo is required for establishing the identities of an early-born block of cells in the mushroom bodies and antennal lobes [16], amongst other lineages [17]. Here we report that the expression of Broad-Z4 apparently provides a molecular marker for the next block of postembryonic neurons. Unlike the early embryonic identity factors, though, Broad is not expressed first in the NB and then passed into this cohort of cells. Rather, it appears in neurons some time after their birth, likely in response to hormonal conditions experienced by them or their parent GMC.

Clonal studies on *Drosophila *embryos suggest that the generation of neuron classes does not abruptly begin with the postembryonic neurons. Although the embryonic lineages are remarkable for the diversity of their early-born neurons, the later-born cells (farther from the neuropil) in many lineages show clustered cell bodies and similar neurite trajectories, indicating that they may constitute a discrete neuronal type [4,44,45]. We propose that these later cells, along with the post-embryonic neurons, should be called the secondary neurons and the term primary neurons should be confined to the initial neurons that are produced during embryogenesis during the progression from Hunchback to Castor. Typically, the first four GMCs produced by a given NB would account for its primary neurons, although, in some lineages, like NB 7-1, the expression of a factor may be prolonged to give two GMCs that produce similar properties [7]. In the ventral CNS, the primary neurons would account for the majority of the neurons present at hatching. Most of the early-born secondary neurons are also functional and contribute to larval behavior, while others show the arrested development described here.

Uncoupling of the terms primary and secondary from embryonic versus postembryonic origins circumvents problems with using these terms in a comparative context. In more basal insects, like grasshoppers, that have direct development, all of the neurons in the ventral CNS are made during embryogenesis [46], and, hence, would be considered as primary neurons if time of birth was the deciding criterion. Similarly, within the insects with complete metamorphosis, neurogenic arrest occurs at different points in the lineage depending on whether you are making a reduced nervous system for a relatively simple larva like a fly maggot, or a complex CNS that is a feature of the hunting larvae of ground beetles [47]. Basing the designation of primary and secondary on the mechanisms that generate cellular diversity, rather than on the stage at their birth or their functional status within the network allows for homologues to have the same designation across these diverse taxa.

### Temporal and spatial features of Br-Z3 expression

In *Drosophila*, most primary neurons express low to moderate levels of Br-Z3 during late embryogenesis. We did not characterize their embryonic expression in detail, and these neurons do not then re-express Br-Z3 (or any other Broad isoform) when they undergo remodeling at the end of larval life ([36] and this study). The secondary interneurons also transiently express Br-Z3 during their early development. The results from feeding larvae on a BrdU diet from hatching (Figure 2) indicate that these embryonic secondary neurons account for many of the strong Br-Z3+ cells seen at hatching.

**Figure 9:**
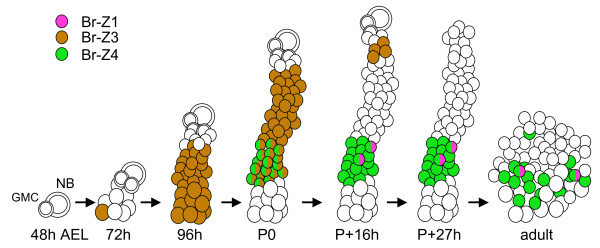
**Summary of the time of the expression of the various Broad isoforms in the secondary neurons of the thoracic segmental lineages**. AEL, hours after egg lay; GMC, ganglion mother cell; NB, neuroblast; P, pupariation.

The main focus of the paper has been on the expression of Broad in the neurons born during the larval phase of neurogenesis (Figure 9). We used BrdU incorporation to measure the latency to the onset of Br-Z3 expression in the secondary neurons. For larvae at 96 h AEL, the BrdU pulse length needed to see labeling in Br-Z3+ neurons was 11 h (Figure 3B, C), and defines the latency from the end of DNA synthesis in a GMC to the appearance of Br-Z3 in its daughters. At 96 h AEL (72 h post-hatching), the average lifetime of a GMC is about 7 h and its G2 phase lasts about 4 h [30]. Hence, a young neuron starts expressing Br-Z3 about 7 h after its birth, which is about 14 h after the birth of its parent GMC. The arrested embryonic NBs resume dividing early in the second instar larva, and GMCs are seen associated with them by 60 h AEL (36 h post-hatching [30]). In this study, we started to see Br-Z3 appearing in the clusters of neurons by about 72 h AEL, a timing consistent with a 14 h latency between the birth of a GMC and the subsequent appearance of Br-Z3 in its daughter cells. Consequently, we assume that this latency is likely similar throughout the period of postembryonic neurogenesis.

Although the secondary neurons may be relatively uniform in the timing of the onset of Br-Z3 expression, they differed in how long this expression was maintained. One pattern was shown by the embryonic and oldest postembryonic secondary cells. These showed initial Br-Z3 expression but then lost it by the start of wandering (Figure 9). They correspond to those cells characterized as being Broad negative and Chinmo+ at pupariation [17]. The other pattern was shown by later born neurons (whose GMCs are born after about 72 h AEL) and these show the persistence of Br-Z3 through pupariation but then lose it early in adult differentiation.

The distinction between the Chinmo+ and Broad+ groups of secondary neurons is set up by the postembryonic actions of Seven-up and Castor [17]. For many lineages, removal of Seven-up resulted in the loss of Broad+ neurons in favor of the larger Chinmo+ cells. A similar result was seen in some of the lineages by the prolonged expression of Castor [17]. It is interesting that both Seven-up and Castor have roles in temporal identity in the embryo [8]. It could be that Br-Z3 expression in the embryonic secondary neurons may likewise be the result of the embryonic interplay of these two genes.

### Broad Z3 and early neuronal development

The appearance of Br-Z3 in thoracic interneurons about 7 h after their birth coincides with the loss of expression of Notch in the membrane of these young neurons (JA and JWT, in preparation). Notch is prominent in the membranes of newborn neurons during pathfinding but then rapidly disappears. Consequently, the appearance of Br-Z3 is correlated with a new neuron arriving at its initial target. The best data on the behavior of growth cones in this post-pathfinding stage are for in-growing retinal axons in the first optic neuropil in *Drosophila *[48]. After arriving at their initial location these axons undergo a 'sorting phase', during which their growth cones spread filopodia laterally to select their cartridge partners. Interestingly, photoreceptors are the only sensory neurons that we have found to express Br-Z3 and they do so during this early sorting phase (data not shown).

Expression of Br-Z3 in the developing medulla interneurons may also be correlated with the sorting of potential partners. As described above, when Br-Z3 first appears in the medulla in the mid third instar, it is confined to the most medial columns of neurons. Through time, more lateral columns progressively acquire Br-Z3 expression, and all outer medulla neurons are expressing this isoform by 14 h APF (Figure 5C). The neurons of the most medial columns interact with the first photoreceptors growing in from the posterior border of the eye [48], and as axons grow in from the subsequent rows of ommatidia over the next 2 days, these interact with the neurons of progressively more lateral columns. The wave of Br-Z3 expression across the medulla therefore matches the wave of afferent in-growth into this structure. Br-Z3 expression in the medulla remains prominent through 27 h APF after which it fades. Throughout this time the axon terminals of photoreceptors R7 and R8 are refining their initial positions in the layers of the medulla [49].

The Br-Z3 expression in thoracic interneurons suggests that there may be a similar 'sorting phase' within the developing central neuropils. The exact role of Br-Z3 in this sorting process is still being explored, but the ectopic expression of Br-Z3 in the LNv neurons (Figure 8) shows that the presence of Br-Z3 during initial outgrowth results in excessive sprouting (Figure 8). If the expression of Br-Z3 in the secondary neurons marks a sorting phase, then groups of these neurons may vary in the time when the sorting is completed. Notably, the early, Chinmo+ secondary neurons lose Br-Z3 expression by the start of wandering (approximately 110 h AEL). This early loss may mean an early commitment to initial targets, and they may serve a pioneer function in organizing the structure of the nascent adult neuropils. The bulk of secondary neurons, however, retain BR-Z3 expression until neurogenesis is almost complete in the central brain and the thorax. The last-born cells in these regions show only a very brief Br-Z3 expression as they are inserting into a neuropil that is already highly sorted.

Br-Z3 expression in the larval γ neurons of the mushroom body is notable because it is the rare case of functioning neurons that continue to express Br-Z3. We do not know the significance of this expression. This may represent a unique function of Br-Z3 in larval mushroom body neurons. Alternatively, it may indicate that a 'sorting phase' may continue even in mature neurons. γ neurons are continually added through the early larval stages and the older γ neurons may have to continue to sort and rewire as the γ class swells in number. Besides the mushroom body γ neurons, we found that a few mature-looking neurons in the brain and thorax of newly hatched larvae were also BR-Z3 positive (data not shown). We do not know the identity of these neurons, but as proposed for the mushroom body γ neurons, they may also have some unique plasticity demands as the larva grows.

A final puzzle involving Br-Z3 is its absence from motoneurons. In the growing larva, the axons from the lineage 15 motoneurons extend to the leg imaginal disc where they end without obvious elaborations. The subsequent interactions of their sprouting axon terminals with nascent muscles may require a very different set of processes from those used by interneurons as they sort out central targets.

### Broad isoforms as markers of temporal identity

The BTB domain transcription factor gene *chinmo *was the first temporal specifying gene found for the postembryonic neurogenic period. Its presence in early-born neurons in both mushroom body and antennal lobe projection neuron lineages establishes early neuronal phenotypes in these lineages [16]. Most recently, Maurange *et al *[17] showed that, just prior to metamorphosis, the secondary neuron lineages can be divided into older, Chinmo+ and younger, Broad+ neurons, the latter being due to the Br-Z3 isoform (this study). Both Chinmo and Br-Z3 disappear early in metamorphosis, though, and it is not known how their transient expression is then turned into persisting markers of temporal identity. We find here that other Broad isoforms, notably Br-Z1 and Br-Z4, provide permanent molecular time-stamps of birthdates.

Prior to metamorphosis, the Br-Z4+ neurons were always found clustered within a given lineage, a pattern suggesting that they arose from consecutively born GMCs. Br-Z4+ neurons were found in all of the thoracic lineages, except for the very small motor lineage generated by NB 24. Although we did not analyze brain lineages in detail, the broad pattern of Br-Z4 expression shows that this isoform is also expressed in most brain lineages. Thoracic lineages that made two classes of interneurons had roughly twice the number of Br-Z4+ neurons as lineages that produced only one class of interneuron (Figure 6E). The secondary neurons generated by a NB can be divided into two hemilineages, each composed of the collective Notch+ or Notch- daughters arising from the division of the series of GMCs. Studies blocking programmed cell death showed that in lineages with one bundle, one of the daughters consistently dies and only one hemilineage survives (DWW and JWT, in preparation). The lineages with both hemilineages had roughly twice the number of neurons compared with those that retain a single hemilineage (Figure 6E). This pattern is consistent with a thoracic NB typically generating 13 to 16 GMCs whose daughters will express Br-Z4, but then cell death reducing the number by half in those that have a single hemilineage. Some lineages may deviate from this pattern; for example, lineage 0 has more Br-Z4+ neurons than would be expected for a lineage with a single surviving daughter, and atypically small lineages, such as lineages 2 and 15, have reduced numbers of Br-Z4+ cells.

MARCM clones induced at 24 and 54 h AEL contained their complete complement of Br-Z4+ cells (Figure 6E, and data not shown). Those induced at 76 h contained only a few Br-Z4+ neurons, and most Br-Z4+ cells were outside of the clone boundaries (Figure 6B, E). The presence of about six Br-Z4+ neurons within the latter clones suggests that only three to six GMCs with the Br-Z4 fate were left to be born at the time of the clone induction. Given that a NB produces about one GMC per hour at this time in larval life [30], we estimate that the GMCs that generate the Br-Z4 cells are born from about 66 to about 80 h AEL (that is, from about 18 h into the second instar until about 8 h of the third larval stage). The analysis of MARCM clones in the adult (Figure 6D) shows that neurons born during this period in the larva continue to express Br-Z4 after metamorphosis. Br-Z4 expression in the adult therefore provides a molecular marker for the neurons whose GMCs were born during this window of larval life. As also described in the Results, Br-Z1 is co-localized in a subgroup of the Br-Z4 neurons, but we do not know if this expression defines a specific temporal subset of the Br-Z4 neurons. Within the postembryonic lineages, neuronal fate is clearly related to the time of birth [11-13]. How Br-Z4 and Br-Z1 participate in these fate decisions remains to be examined.

*Broad *differs from the embryonic temporal specification genes in that neither Br-Z4 nor Br-Z1 is expressed in the NBs or parent GMCs (data not shown). Br-Z4 expression only becomes evident towards the start of metamorphosis, well after the neurons are born. This suggests a mechanistic difference in the way in which primary versus secondary neurons are 'time-stamped'. The cells that will express Br-Z4 at the outset of metamorphosis are included in the Br-Z3+ group and not in the Chinmo+ group. Based on its timing, the Chinmo/Broad-Z3 boundary likely marks the start of production of the Br-Z4 neurons. Interestingly, Chinmo and Br-Z3 then disappear early in metamorphosis, whereas Br-Z4 persists as a permanent temporal marker for these neurons. This is the first reported example of a marker for temporal identity that persists into the adult.

A second potential difference from the embryonic pattern is in the signals that control expression of the timing genes. In the embryo, the switch from one timing gene to the next is intrinsically controlled within each lineage [8]. In the postembryonic phase of neurogenesis, by contrast, extrinsic factors appear to play a role in determining the timing of switching from one cell type to another (E Marin and JWT, unpublished). The switching on and off of GMCs that will make Br-Z4+ neurons coincides with two 'milestones' in *Drosophila *larval development. The GMCs with the Br-Z4 fate start to be made late in the second instar, around the time larvae achieve the threshold size for metamorphosis and become committed to entering the terminal larval stage [50]. They stop being made at the time in the third instar when the larva achieves its 'critical weight' sufficient to carry it through to pupariation and metamorphosis [51]. Both of these milestones represent endocrine-generated decisions. These extrinsic signals may be responsible for the expression of Br-Z4 and the specification of a distinct subtype of neuron, but this remains to be examined.

## Materials and methods

### Fly stocks

Flies were reared on the standard yeast-cornmeal-molasses diet. The Canton-S stock was used as a 'wild-type' stock for experiments. The *nonpupariating lethal *(*br*^*npr*3^) was used as a null mutation that eliminates expression of all Broad isoforms [26]. The *elav*^*C*155 ^GAL4 driver is an enhancer trap insertion into the *elav *locus (*P{GawB}elav*^*C*155^) [52], and this was used to generate clones in the CNS using the MARCM technique [53]. Females of the genotype *P{GawB}elav*^*C*155^, *hsFLP*; FRT42B, *tub-GAL80*/*CyO *were crossed to males that were FRT42B, *UAS-mCD8::GFP*. For the embryonically induced clones, eggs were collected for 2 h, held for 3 h at 25°C, and then given a 1 h heat shock at 37°C (that is, the embryos were heat shocked between 3 and 5 h of embryogenesis). For inducing clones early in larval life, we typically collected eggs over a 1 to 2 h period, maintained them at 25°C for the appropriate amount of time after egg laying (for example, 24 h to obtain newly hatched larvae), and then heat-shocked the larvae at 37°C for 45 minutes to 1 h.

During division of the NB, two different types of green fluorescent protein (GFP)-expressing clones can be produced depending on whether the NB or the GMC loses the GAL80 suppressor [53]. We analyzed both NB clones (which include the remainder of the lineage) and GMC clones (which are the pair of neurons from the GMC division).

### Construction of the UAS-BR-Z3 transgenic line

A cDNA clone that encodes the *Drosophila *BR-Z3 isoform (dm797, a gift from Dr L von Kalm) [54] was cloned into the UAS vector *pUAST *(gift from Dr CS Goodman). The transformation construct (300 to 500 μg/ml) and the helper plasmid pTURBO (100 μg/ml) were dissolved in injection buffer (5 mM KCl, 0.1 mM phosphate buffer, pH 7.8). The mixture was injected into embryos of the *w*^1118 ^stock. Transformants were selected as flies with reddish eye color among the progeny of individual injected flies. Isolation of homozygous insertion lines was achieved by performing standard genetic crosses with the marked balancer stock *y w; Bc Elp/CyO; Ki/TM3*, *Sb*.

### Immunocytochemistry and *in situ *hybridization

Collection, dechorionation and fixation of embryos were performed as described in [55]. Nervous systems were dissected from animals at various stages and fixed in buffered 3.7% formaldehyde for 30 minutes at room temperature and then washed 3 times in PBS-TX (phosphate buffered saline (pH 7.8) with 1% Triton-X100). Fixed samples were blocked in 2% normal donkey serum (Jackson ImmunoResearch Laboratories, West Grove, PA, USA) in PBS-TX for 30 minutes and then incubated in various combinations of primary antibodies for 1 to 2 days at 4°C. Primary antibodies were used at the following concentrations: 1:250 mouse anti-Broad core MAb [54] (gift from Dr G Guild); 1:3,000 rabbit antisera against each of the BR-Z isoforms [56] (gift from A Antoniewski and J-A Lepesant); 1:1,000 rabbit anti-β-galactosidase (Invitrogen, Carlsbad, CA, USA); 1:1,000 rat anti-elav (gift from Dr S Robinow); 1:20 mouse anti-neurotactin MAb (F4A, gift from Dr M Piovant); 1:1,000 rat anti-mCD8 (Caltag Laboratories, Burlingame, CA, USA). After three to four rinses to remove the primary antisera, tissues were generally incubated overnight in combinations of fluorescein isothiocyanate (FITC)-conjugated and Texas Red-conjugated secondary antibodies at 1:500 dilution (for example, FITC-conjugated donkey anti-rabbit IgG, and Texas Red-conjugated anti-mouse IgG) (Jackson ImmunoResearch Laboratories). Nervous systems were then rinsed, mounted on poly-lysine coated coverslips, dehydrated through an ethanol series, cleared in xylene and mounted in DPX (Fluka, Bachs, Switzerland). Non-fluorescent detection employed horse radish peroxidase-conjugated secondary antibodies, in conjunction with peroxidase-peroxide stimulated precipitation of a colored polymer of diaminobenzadine (Sigma, St Louis, MO, USA).

### BrdU labeling

We used the incorporation of BrdU to identify cells that had undergone DNA synthesis [57]. For chronic labeling of all of the neurons born during larval growth, we fed larvae on a diet containing 0.1 μg BrdU/ml as described in [30]. The same concentration was used for the phasic studies to determine the latency to Br-Z3 expression. At the end of the labeling period, larvae were dissected and their CNSs fixed in buffered formaldehyde for 30 minutes to 1 h. Labeled DNA requires acid hydrolysis prior to detection by anti-BrdU immunocytochemistry [50], but this treatment can destroy other epitopes in multiply immunostained preparations. Consequently, we typically treated the tissues as described above with primaries and then secondaries for the non-BrdU epitopes. The incubation in secondary antisera was followed by rinses and then DNA hydrolysis in 2N HCl in PBS-TX for 1 h. Tissues were then rinsed and then exposed to a 1:100 dilution of an anti-BrdU monoclonal antibody (Becton-Dickinson, Franklin Lakes, NJ, USA) for three nights followed by the fluorescently tagged anti mouse IgG. Tissues were dehydrated, cleared and mounted as above.

## Abbreviations

AEL: after egg laying; APF: after puparium formation; Br: Broad; BrdU: bromodeoxyuridine; Br-Z: Zinc finger isoform of broad; BTB: broad-tramtrack-brick-a-brac; CNS: central nervous system; GFP: green fluorescent protein; GMC: ganglion mother cell; IR: immunoreactivity; MARCM: mosaic analysis using a repressible cell marker; NB: neuroblast; PBS: phosphate buffered saline; PBS-TX: phosphate buffered saline with TritonX 100.

## Competing interests

The authors declare that they have no competing interests.

## Authors' contributions

BZ initiated the study, established early expression patterns and wrote the first draft of the manuscript. DW participated in the MARCM clone production and analysis. JA carried out the birth-dating studies and some of the Br-Z3 analysis. LR participated in the design and coordination of the study and helped to draft the manuscript. JT carried out some of the immunocytochemistry, the Br-Z4 analysis, and coordinated the final draft of the manuscript. All authors read and approved the final manuscript.
